# Supplementary material for: “I Think That’s the Most Beneficial Change That WIC Has Made in a Really Long Time”: Perceptions and Awareness of an Increase in the WIC Cash Value Benefit
Source: Int J Environ Res Public Health. 2022 Jul 16;19(14):8671. doi: 10.3390/ijerph19148671 (PMC9324401; doi:10.3390/ijerph19148671)
Supplement: Supplementary file 1 [file ijerph-19-08671-s001.zip › Supplemental FIle S1.pdf]

Supplemental File S1: Focus group guide.

[Note for IRB: This is a semi-structured focus group guide so the focus group facilitator may adapt some of the language below during the focus group and use spontaneous probing.]

Aims:

- Examine Special Supplemental Nutrition Assistance Program for Women, Infants, and Children (WIC) participant facilitators and barriers to utilizing the increased cash value benefit (CVB) for fruits and vegetables
- Assess participant awareness of the change in the increased CVB
- Examine WIC participant perceptions of how the increased CVB has affected their household's food security and dietary behaviors
- Examine how WIC participants perceive that COVID-19 affected their food security and food-related behaviors

Key:

Text that is read out loud by interviewer to participant

[Instructions to interviewer; text not read to participant]

1. *Probes for interviewer to use as needed*

Introduction

Thank you all very much for joining us today, and for being patient as we set everyone up. My name is --- and I'll be leading our discussion today. I also have my co-worker(s) here, --- and ---. They will be helping with the technology and taking notes, but they won't be participating in our discussion. We are part of a research team at The University of North Carolina at Chapel Hill. Before we get any further, are there any questions about the consent form that we sent you via email?

We are having these group discussions because we are interested in your experiences with a recent increase in the amount of WIC benefits provided to purchase fruits and vegetables.

Our discussion should last about 60 minutes, and it will be audio and video recorded. We encourage you to leave your cameras on because this will allow us to have a more meaningful discussion. If you need to turn off your video because of internet speeds or connection issues, we understand. Related to turning off your cameras, we also want to let you know this is a safe space if you need to pump or breastfeed you can certainly turn off your camera as needed.

We want to make sure we provide a welcoming space for you to participate. I will be here to listen and ask some specific questions. You all are the experts on this topic, so we want to hear from you about your experiences and ideas. There will be no judgement of your responses or answers. I want to make sure you all know that we do not work for WIC, we are employees of UNC, and your responses will not affect your participation in the WIC program in any way. Feel free to make any positive or negative comments about the things we will be discussing today. We want this to be a free-flowing discussion and there are no right or wrong answers.

To be respectful of your time, I may need to interrupt at times to move us forward. Thank you for your understanding if I need to do this.

Because I am here to listen, there may be long pauses between your responses as I hold space to make sure you have felt heard and shared all you wanted to share.

Before we start, there are some points I'd like to go over.

We want to encourage everyone to actively share in the conversation. Everyone has had different experiences that we want to hear. But also, be aware of how much you're speaking. If you are talking a lot, I may interrupt you, and if you aren't saying much, I may call on you. If I do, please don't feel bad about it. It is just my way of making sure we get through all the questions and that everyone has a chance to talk.

I also wanted to let you all know that everything that you say here will be kept anonymous. Nothing said in this group will ever be associated with any individual by name. We would also ask that you similarly not associate anything said in this group with anyone by name.

We also want to make sure you feel as comfortable as possible sharing, so if you would like to change your name to an alias you can do so by clicking on Participants, hovering over your name, clicking "Rename" and then typing in a new name. Additionally, if there is something you don't feel comfortable sharing out loud with the group you can use the chat function.

The information that you share with us today will be written up in an article to be published in a scientific journal. It will also be shared with the National WIC Association and the state WIC office so that our findings can inform improvements to the WIC program, so your input is very important. We also plan to share a summary of our results with you all in a few months when the results are ready.

As we are talking today, I also want to encourage you to show agreement or disagreement in the chat or verbally. So we can keep the conversation moving, if you agree or disagree with something another participant is saying and would like to let us know, you can type a short message in the chat window or you can say you agree or disagree.

If you accidentally exit or have connection issues, please do your best to re-enter. You can do so by clicking on the same Zoom link you used to get in the first time. If you're having trouble, you can also contact ---, who is assisting with the technology for this interview at XXX-XXX_XXXX [will use a google voice phone number].

[Put phone number in the chat for the participant]

Okay great, before we get started, let's briefly go around and introduce ourselves. Please tell us your first name, or if you do not wish to share your name you can use an alias, and your favorite time of year or season and why. I'll start. My name is --- and my favorite time of year is --- because ----. **[moderator then "calls" on people to quickly introduce themselves].**

Thanks for introducing yourselves.

Do you have any questions before we get started? Great, now we'll dive into the conversation.

[Start audio recording: zoom]

General shopping and WIC utilization behaviors:

Let's start by just talking a little bit about the WIC program, your general shopping behaviors, and how you use your WIC benefits.

1. Tell me about your process getting enrolled in the WIC program.
    a. Is there anything you would like to see changed about the enrollment process?
2. Tell me about your grocery shopping experiences with WIC over the last 30 days. For example, where did you go? What stores do you like using your WIC benefits at and why? How often did you go grocery shopping?
3. How do you decide on which items you use your WIC benefits on? [*Probe what foods parts of the food package that participants like and what parts of the food package they wish were different*]

COVID and food acquisition behaviors

Now we'd like to hear a little bit about your experiences with providing food for your family during the pandemic.

1. How, if at all, did you change how you shopped for groceries during the pandemic? [*Probes for changes in types of foods/beverages, shortages, frequency of trips, going to fewer/more stores, changes in food cost, shopping online, differences between early on in the pandemic and now, certain food categories (e.g. FV) that were easier/more difficult to buy]*

2. Similarly, think back over the course of the pandemic, how, if at all, have you changed how you used your WIC benefits throughout the month? [*Probe for things that made it easier or more difficult to use WIC benefits like shortages, flexibilities in the WIC food package*]

Awareness of and access to FV benefit changes

1. In June of 2021, the amount of WIC benefits to buy fruits and vegetables increased from about $10/person to $35/person each month. What did you know about this change, if anything? [*Probes if they say they were aware of it- How did you find out about this change?*]

    5a. In September of 2021, the benefit then decreased for one month back to $10 and then increased again in November. What did you know about this change, if anything? How did you find out about it? Did this cause you any difficulties?

2. How are you currently getting information about changes to the WIC program such as this recent change in the amount for fruits and vegetables? How would you like to get information about changes to the WIC program [*Probe/examples: via text message, on the app, from a WIC staff person]*?

3. How did you feel about the changes in the amount of money for fruits and vegetables? What did you like most about these changes? What was frustrating or what did you like the least about these changes? [*Probe: was the increase something that met their household's need or would something else have been more helpful*? *For those participating pre/post policy change- was the old amount sufficient? Do they need more than the current amount? How has the change in food prices and availability due to the pandemic affected redemption if at all?*]

Shopping experience during benefit change

[If someone in the group did not know about the program change, you can ask them about what they hypothetically would do with more money for fruits and vegetables or direct questions at participants that were aware of these changes.]

1. With the new fruit and vegetable benefit amount- about how much of the amount available for fruits and vegetables would you usually spend in a month? [*Probes Were there things that made it easy to spend the full amount? Were there things that made it hard to spend the full amount?*]

2. How, if at all, do you think having more money for fruits and vegetables affected your other purchases at the grocery store? [*Probes Were there other foods or beverages that you bought more of because you had this benefit? Were there other foods or beverages that you bought less of because you had this benefit*?]

3. How did you feel about the increased benefits only being allowed for fruits and vegetables? [*Probe about if they would have preferred the benefits be used for other foods in the WIC package or just other foods more generally and why?]*

4. If you had trouble spending your whole FV allotment, what kinds of things would make it easier for you to use all your fruit and vegetable money from WIC each month?

Food behaviors during benefit change

4. In a typical week when you had more money from WIC for fruits and vegetables, what types of meals would you and your family eat? Was this similar to or different from what you would usually eat? How? [*Probe for buying different types of fruits and vegetables, allowing children to try new fruits and vegetables, amount of food thrown away before it could be eaten or because family did not like it]*

Perception of the value of participating in WIC

5. How, if at all, did this increased FV benefit influence your decision to remain enrolled in the WIC program?

6. If the benefit amounts go back to $9-11/month/person, would that influence how likely you would be to continue to participate in WIC?

Closing

So, that brings us to the end of the discussion. Is there anything that hasn't already been raised that you think is important to include or mention?

[Turn off recording]

Thank you all very much for your time. We are grateful for your willingness to share your thoughts and feelings with us. If you have any further thoughts to share, please e-mail us at [insert study email]. We will be reaching out to you all soon with some information about local food and nutrition programs and common eating challenges among young children in case you are interested, as well as your gift card. We are trying our best to process the gifts cards as quickly as possible, but please note that it may take one to two weeks.
