# Supplementary material for: “I Think That’s the Most Beneficial Change That WIC Has Made in a Really Long Time”: Perceptions and Awareness of an Increase in the WIC Cash Value Benefit
Source: Int J Environ Res Public Health. 2022 Jul 16;19(14):8671. doi: 10.3390/ijerph19148671 (PMC9324401; doi:10.3390/ijerph19148671)
Supplement: Supplementary file 1 [file ijerph-19-08671-s001.zip › Supplemental Table S1.pdf]

Supplemental Table S1. Codebook including codes, subcodes, definitions, and examples of concepts to include and exclude.

Code/subcodes	Definition	Inclusion	Exclusion
**Cash Value Benefit**			
Awareness	Notified by WIC of changes to CVB amount including initial increase and subsequent changes	Participant received a notification (email, text, phone call) from their local WIC agency about the initial CVB increase or any future changes	Participant found out about CVB increase from checking WIC app for benefit balance
Lack of awareness	Not notified by WIC of changes to CVB including initial increase and subsequent changes	Participant found out about CVB increase or future changes through some method other than local WIC agency	Participant found out about CVB change from local WIC agency
Information source	Resources identified by participants that inform them of changes within WIC, including CVB changes	Communication from WIC, websites, letters, flyers, emails, social media	Preferred information sources
BNFT App	Finding out about the CVB changes through the BNFT app	Mentions finding out about CVB changes through the BFNT app	Finding out about the change through another source
Receipt	Finding out about the CVB changes on their grocery store receipts	Mentions finding out about CVB changes by looking at grocery store receipt	Finding out about the change through another source
Call	Finding out about the CVB changes from a phone call from WIC staff	Mentions finding out about CVB changes by receiving a call from local WIC agency	Finding out about the change through another source
Text	Finding out about the CVB changes from a text from WIC staff	Mentions finding out about CVB changes by receiving a text from local WIC agency	Finding out about the change through another source
Email	Finding out about the CVB changes from an email from WIC staff	Mentions finding out about CVB changes by receiving an email from local WIC agency	Finding out about the change through another source
Preferred information source	Favorable communication channels and platforms for changes within WIC, including CVB changes	Methods of communication that WIC participants prefer for updates from WIC about changes	Method of communication used for CVB change
Call	Preferring to receive a call from WIC when there are changes	Preferring to receive a call from WIC when there are changes	Preferring other sources

Text	Preferring to receive a text from WIC when there are changes	Preferring to receive a text from WIC when there are changes	Preferring other sources
Email	Preferring to receive an email from WIC when there are changes	Preferring to receive an email from WIC when there are changes	Preferring other sources
App	Preferring a BNFT app notification when there are changes	Preferring to get a BNFT app notification when there are changes	Preferring other sources
Timing met needs	The CVB increase in June 2021 met the households' needs at that time and they did not need other components of the food package changed at that time	References to the CVB increase in June 2021 meeting households' needs at that time	References to using CVB first each month, saying CVB is the most valuable part of the WIC food package
Timing did not meet needs	Needing some other WIC food package component (other than CVB) increased in June 2021	References to needing some other WIC food package component (other than CVB) increased in June 2021	General references to desired changes to other components of the WIC food package
Challenges of temporary CVB decrease	Difficulties associated with the CVB decrease from $35 to $9-11/person/month in October 2021	Any difficulties with planning, shopping, or meeting household food needs experienced in October 2021 when the benefit decreased temporarily or negative feelings about this decrease	Lack of awareness of the CVB decrease in October
Benefits or positive perceptions of CVB increase	Positive aspects of larger CVB amounts (either $35/month or NASEM amounts)	General positive perceptions of the CVB increase at the initial $35/month or NASEM amounts	Positive perceptions of other WIC food package components or COVID-related changes to WIC
Variety	Perceived changes in the types of fruits and vegetables purchased and consumed	New types of fruits and vegetables, different forms (i.e., fresh instead of frozen) of fruits and vegetables	Purchasing or eating more (volume not types) of fruits and vegetables generally
Family/mother/ caregiver diet	Perceived improvements in the eating habits of family members other than the child enrolled in WIC	Mentions of parents or the whole family eating healthier because of the increase in fruits and vegetables	Mentions of eating more variety
Child diet	Perceived improvements in the eating habits of children enrolled in WIC	Mentions of the child eating healthier because of the increase in fruits and vegetables, child snacking on fruit or eating fruit for breakfast, eating more fruits and vegetables	Mentions of the child eating more variety

	Engaging children in shopping	Mentions of children selecting new fruits and vegetables or talking about fruits and vegetables in the grocery store environment	Descriptions of child helping parent select new fruits and vegetables in the grocery store after the CVB increase	General references to increase in variety of fruits and vegetables or eating new fruits and vegetables
	Achieve family's/culture's dietary preferences	Being able to access foods that meet families preferred dietary behaviors/patterns (e.g., vegan, Latina, organic)	Describing being able to provide meals in line with preferred diet after the CVB increase such as vegan diets, purchasing organic, having more money to purchase more expensive, WIC ineligible foods such as meat	Other components of the WIC food package that do/do not align with household's food culture
	Food waste	Being less afraid to waste food when offering new fruits and vegetables to kids	Being less afraid after the CVB increase to waste food when offering new fruits and vegetables to kids	References to wasting foods other than fruits and vegetables or wasting components of the food package other than fruits and vegetables
Uncertainty about CVB amounts		Feeling uncertain about how long the CVB increase will remain in effect or changes in the CVB amount over time creating uncertainty	Lack of clarity about how long the CVB change will remain in place, uncertainty about the CVB amount participants should be receiving currently	Negative aspects of the October dip in benefits in NC
Amount of CVB used		The amount in terms of dollars or percentage etc. of the total CVB value that each participant uses each month and/or how many trips to the store the CVB lasts for	Any reference to the amount of the monthly CVB allotment used by participants and/or how many trips to the grocery store the CVB lasts for	Mentions of the amount or percentage of the total WIC food package used each month
Satisfaction with CVB amount		General approval or appreciation of having CVB for fruits and vegetables	Approval or satisfaction of either the old or new CVB amounts	Not liking the old or new CVB amounts
	Old CVB	Contentment with and acceptance of $9-11 CVB pre-COVID	Approving of the $9-11/person/month CVB	Approving of the $35/person/month or NASEM amounts
	New CVB	Contentment with and acceptance of new CVB amounts	Approving of the $35/person/month or NASEM amounts	Approving of the $9-11/person/month CVB
Dissatisfaction with CVB amount		General disapproval of having CVB for fruits and vegetables	Disapproval or dissatisfaction of either the old or new CVB amounts	Approving of the old or new CVB amounts

	Old CVB	Discontentment with or disapproval of $9-11 CVB pre-COVID	Disapproving of the $9-11/person/month CVB	Negative aspects of the October dip in benefits in NC
	New CVB	Discontentment with or disapproval of NASEM CVB amounts compared to the initial $35/month	Mentions of challenges associated with or disapproval of decreasing from $35/month to the NASEM amounts	Negative aspects of the October dip in benefits in NC
Facilitators of using CVB		Factors that make it easy to redeem the full CVB amount each month	Variety of fruit and vegetables options within CVB, supply in stores, preferences for fruits and vegetables, or other facilitators that make it easy to spend the full CVB amount	General facilitators to using WIC while grocery shopping
	Variety of products offered/accepted	The variety of products that are approved through WIC CVB being a facilitator to spending CVB	Mentioning the many options for fruits and vegetables that can be purchased with CVB	Increasing variety of fruits and vegetables purchased or consumed after the CVB increase
	Retail type	Access to sufficient fruits and vegetables in the store environment being a facilitator to spending CVB	Types of retail (either specific stores or formats) where it is easier to use and redeem CVB and sufficient supply of fresh and appealing fruits and vegetables	Store types where WIC participants generally like to use WIC benefits, mentions not specific to using CVB
	Household preferences	Household preferences for consuming fruits and vegetables being a facilitator to spending CVB	Mentions of families liking fruits and vegetables or that being part of their food culture and those things making it easy to use the full CVB amount	Mentions of household preferences related to other foods than fruits or vegetables or other WIC food package components
Barriers to using CVB		Factors that make it hard to redeem the full CVB amount each month	Unclear fruit and vegetable labeling, issues identifying eligible fruits and vegetables in the app, issues at checkout, insufficient supply or time to prepare fruits and vegetables, having too much for fruits and vegetables in combination with other incentive programs, or other barriers to using the full CVB amount	General barriers to using WIC while grocery shopping
	Unclear labeling	Unclear labeling of WIC eligible fruits and vegetables	Only references to unclear labeling of fruits and vegetables	Unclear labeling of other WIC approved items

BNFT app	Inability to use the BNFT app to determine if some fruits and vegetables are WIC approved	References to the BNFT app not working well for fruits and vegetables	Other mentions of issues with the BNFT app not specific to fruits and vegetables
Issues at checkout	Issues at the checkout stage with purchasing seemingly WIC approved fruits and vegetables	References to issues at checkout specifically related to fruits and vegetables and the CVB	Other issues at checkout related
Retail type	Insufficient access or supply of appealing fruits and vegetables at certain stores or store types that participants like to purchase fruits and vegetables at not accepting WIC	Mentions of stores or retail types where people like to buy produce that do not accept WIC (e.g., ALDI) or mentions of stores they do shop at not having sufficient supply of appealing fruits and vegetables	Store types where WIC participants generally do not like to use WIC benefits, mentions not specific to using CVB
Time to prepare food	Having insufficient time to prepare fruits and vegetables in meals due to other demands	Insufficient time to prepare fruits and vegetables as a barrier to fully utilizing the CVB	Mentioning time to prepare food as barriers to using other WIC food package components other than the CVB
Proposed changes to CVB	Recommendations for different amount for CVB, eligibility for CVB, or to improve use of CVB amount	Allowing 6+month olds to receive CVB instead of baby foods, statements about needing more than the current CVB amount	Proposed changes to other aspects of the WIC food package
Strategies to stretch CVB	References to using certain strategies or buying certain types of fruits or vegetables to maximize the amount of CVB available	Mentions purchasing bags of apples or oranges because of low cost and shelf life, only purchasing bananas because of low cost per oz	Mentions of increased variety now that the CVB is higher
Value of the CVB relative to other WIC food package components	Stating that the CVB is the most valuable component of the WIC food package, a reason for remaining in the WIC program, or that participants use their CVB first before other components of the WIC food package	Mentioning the CVB as a reason to remain enrolled in WIC, mentioning using CVB first before other WIC benefits, CVB being favorite component of food package	General benefits of participating in the WIC program
**WIC Benefit Utilization Behaviors**			
Utilization Facilitators	Aspects that make it easy for WIC participants to use WIC benefits while grocery shopping	Clear labels, BNFT app working well	Poorly labeled items, issues at checkout, BNFT app not working

	Clear labeling	Clearly and accurately labeled WIC approved products	Products accurately and clearly labeled as WIC approved	Discussion about poorly labeled products
	Store preferences	Specific stores that participants prefer to redeem WIC benefits in for a variety of reasons	Stating the participant prefers to use WIC at a specific store because they have accurate labeling of WIC products, easy checkout, friendly staff, etc.	Stores that WIC shoppers do not like because of labeling, staff, environment. Discussion of stores participants wish were WIC approved. Preferring a store because it is close to home or has lower prices (things not related to using WIC).
	WIC BNFT App	Use of the WIC BNFT app to know which products are WIC eligible or overall benefit amounts	App functioning well to help participants identify WIC eligible products or know how many benefits they have left	Issues with the app not working
	EBT transition	Transitioning from paper vouchers to EBT cards facilitating use of WIC benefits	Mentions of how the process of using WIC is easier now after the transition to EBT from paper	Mentions of EBT meaning SNAP, issues or negative aspects of WIC EBT
Utilization Barriers		Aspects that make it difficult for WIC participants to use WIC benefits while grocery shopping	Issues at checkout, poorly labeled products, store environment, mental or time burden of using WIC compared to other forms of tender	Facilitators to using WIC benefits in the store, changes participants want to see in the food package
	Unclear labeling	Lack of clear labels for WIC approved products	Unclear, inaccurate, or nonexistent labels for WIC approved products	Clear and accurate labels for WIC approved products
	Checkout experience	Issues at checkout with using WIC benefits	Mentions of items participants thought were eligible not being eligible, having to pay out of pocket or with SNAP for products participants thought were WIC approved, having to wait a long time to use WIC benefits because they cannot use the self-checkout lines	Issues at checkout during the October dip, self-checkout
	Store preferences	Specific stores that participants do not like to use their WIC benefits at for a variety of reasons	Stores that do not have clear labels, easy checkout, friendly staff	Mentions of these topics without reference to a specific store. Discussion of stores participants wish were WIC approved.
	WIC BNFT app	Issues with using the WIC app to identify approved products or benefit levels	BNFT app not working in store, BNFT app not accurately identifying WIC eligible products	BNFT app not working for fruits and vegetables specifically

Mental or time burden	Having to think about which products are approved and using coping strategies to avoid this or having to go to multiple outlets to redeem WIC benefits	Strategies used in store to remember which items are WIC approved, mentions of the challenges of identifying WIC approved items in store, traveling to more than one store to find WIC approved items	Inaccurate or nonexistent labels of WIC approved products, general mentions of food shortages
Stigma	Feelings of shame or social unacceptability of receiving WIC benefits or services	Being embarrassed using WIC benefits, not wanting other shoppers to have to wait for them using WIC benefits	Other issues at checkout not related to feeling embarrassed or ashamed
Desire for new technologies	Wanting to use WIC benefits at self-checkout, using WIC during online grocery shopping, new systems in place at checkout	Mentions of how using self-checkout or being able to use WIC online would make it easier to use WIC benefits, changing grocery store systems to be able to use WIC to leverage deals like buy one get one free	Discussions about using other tender types online or at self-checkout
Desired stores	Stores participants would like for WIC benefits to be accepted	Mentioning other stores like ALDI or Sam's club where WIC benefits cannot be redeemed but participants would like to use WIC	Stores that already accept WIC that participants like or dislike
Frequency	How many trips participants take to use their WIC benefits	Mentions of how many trips participants take to use their WIC benefits	Changes in frequency due to COVID
**WIC Food Package**			
Favorable perceptions of component other than CVB	Any positive or favorable perception expressed about components of the WIC food package except for the CVB	Aspects of the WIC food package participants like, references to food preferences, using more of certain components, how these components fit into family's diets	CVB fruits and vegetables
Juice	Approved 100% juice products	100% juice	Fruits or vegetables
Dairy products	Items derived from cow's milk or non-dairy milk alternative	Milk, cheese, yogurt, soy milk	
Grains/cereal	Approved grains in WIC food package	Breads, hot and cold cereals, rice, pasta, tortillas	Infant cereals
Protein foods	Foods high in protein that are part of WIC food package	Beans, lentils, tofu, fish, eggs, peanut butter	Chicken, beef, pork, seafood other than fish, infant meats
Eggs	Eggs approved in the WIC food package	Eggs	

	Infant foods	Specially formulated products for infants a part of WIC food package	Formula, infant fruits and vegetables, infant cereals, infant meats	
Unfavorable perceptions of component other than CVB		Any negative or unfavorable perception expressed about components of the WIC food package except for the CVB	Aspects of the WIC food package that participants do not like, references to food preferences, how these components do not fit into family's diets	Negative feelings about the CVB, desired changes to the WIC food package, saying they get too much or too little of something (should go under amount)
	Juice	Approved 100% juice products	100% juice	Fruits or vegetables
	Dairy products	Items derived from cow's milk or non-dairy milk alternative	Milk, cheese, yogurt, soy milk	
	Grains/cereal	Approved grains in WIC food package	Breads, hot and cold cereals, rice, pasta, tortillas	Infant cereals
	Protein foods	Foods high in protein that are part of WIC food package	Beans, lentils, tofu, fish, eggs, peanut butter	Chicken, beef, pork, seafood other than fish, infant meats
	Eggs	Eggs approved in the WIC food package	Eggs	
	Infant foods	Specially formulated products for infants a part of WIC food package	Formula, infant fruits and vegetables, infant cereals, infant meats	
Amount of full benefit		How long WIC benefits last each month and whether the amount offered is sufficient	References to the adequacy of the entire WIC food package to meet households' needs	References to the adequacy of the CVB component
SNAP EBT usage		Discussion of using SNAP/EBT in addition to WIC or as a supplement to WIC	Any reference to the role that SNAP/EBT provides in households meeting their grocery shopping needs	References to the WIC EBT card
Desired changes to WIC food package other than changes to CVB		Items participants would like more or less of in WIC food package, items that participants would like to be included in package, and changes in the administration of the WIC food package	Items to reduce or increase in the food package, items to add, differences in how benefits are administered	Unfavorable perceptions of the existing food package, desired changes to the CVB
	Household dietary preferences	More flexibilities in foods received to meet families' eating	Flexibilities or changes to meet households' diverse dietary preferences,	Foods in the WIC food package households like generally, saying they want more or less of some

		patterns (vegan, lactose intolerant, organic)	dietary restrictions, or needs related to food allergies or intolerances	component without additional context related to household preferences
	Substitutions	Substitutions within and across categories to meet needs not related to family's cultural patterns of eating or family's food restrictions	Discussion of substitutions like yogurt instead of milk or bottled water instead of juice	Substitutions related to fruits and vegetables (i.e. more CVB instead of baby food)
	Rolling over benefits	Allowing WIC benefits remaining at the end of the month to roll over to the next month	Rolling over WIC benefits from one month to the next to meet households' needs	Changes in benefit amounts, fear of forgetting to use benefits
	Package sizes	Flexibility in existing allowable package sizes for certain components of the food package and allowing different formats like juice boxes, individually packaged yogurts etc.	Using WIC for different package sizes to make it easier to find products or different serving styles (i.e., individually packaged items)	Shortages of package sizes during COVID
	Eligibility	Desired changes to who is eligible to receive food through WIC	Desired changes to WIC eligibility for the WIC food package e.g., people who breastfeed longer than 1 year, people whose children are on WIC	Barriers to enrollment
	Amount	Statements about desired changes to the amount of specific components of the WIC food package received	Wanting more formula for partially breastfeeding moms, wanting less milk	Statements about wanting different package sizes or formats (e.g., flexibility in package sizes or offering multipacks)
	Disconnect with nutrition advice	Feeling that the food package or components of the food package do not align with the nutrition education delivered by WIC	Nutrition advice given by WIC nutritionists being unattainable given the components of the WIC food package or the foods beings received from WIC not aligning with advice given by nutritionists	General mentions of nutrition advice from WIC
**COVID-19**				
Change in frequency		Any general changes to shopping frequency pre-pandemic to during pandemic	Changes due to the pandemic in the frequency of grocery shopping	General discussions of shopping frequency

	More frequently	Increase in how often participants went grocery shopping	Mentions of increasing the frequency of shopping during COVID	Mentions of change in frequency not related to COVID
	No change	No change in how often participants went grocery shopping	Mentions of no change the frequency of shopping during COVID	Mentions of change in frequency not related to COVID
	Less frequently	Decrease in how often participants went grocery shopping	Mentions of decrease the frequency of shopping during COVID	Mentions of change in frequency not related to COVID
Barriers to food access		Factors that made certain foods, WIC approved or not, more difficult to buy during the COVID-19 pandemic	Shortages, prices, fear, loss of income	General barriers to using WIC benefits not related to the pandemic
	Shortages	Limited or no availability of specific food products in grocery stores/food retail outlets during the COVID-19 pandemic	Lack of availability of foods, WIC approved or not due to the pandemic or during the time of the focus groups (which was during the pandemic)	General discussions of limited availability of foods like fresh produce
	Inflation	Increases in food prices during the COVID-19 pandemic	Discussion of the cost of food increasing during the pandemic	General comments about food cost, not relating to cost increasing
	WIC online shopping	Issues with accessing food or fear of accessing food related to WIC not being accepted for online grocery shopping	Any comments about wanting to be able to use WIC for online shopping during COVID and any issues/increased risk of exposure related to that	Comments about online shopping with other tender types such as SNAP
	Difficulties coordinating childcare	Having to find someone to watch their children to go grocery shopping or the inconvenience and/or fear of taking small children grocery shopping during the pandemic	Mentions of challenges related to finding someone to watch children when parents needed to go grocery shopping in person or the fear of taking their small children in a grocery store to redeem WIC benefits	Other mentions of non-shopping related grocery challenges
	Food insecurity related to job loss/change	Job loss or underemployment leading to food or nutrition insecurity	Experiencing food insecurity or job loss during the pandemic that made it hard to get sufficient food	Experiences with food insecurity or job loss not during the pandemic
Facilitators to food access		Factors that made certain foods easier to buy	Changes in WIC benefits and food package flexibilities, store inventory	General facilitators to using WIC, not specific to during COVID
	WIC flexibilities	Flexibilities in the WIC food package implemented during COVID-19	Changes to the sizes or varieties (e.g., organic) in foods that were WIC approved during the pandemic	Desired changes to the WIC food package or changes in package sizes or variety that occurred outside of the pandemic

Online shopping	Online grocery shopping using payment methods other than WIC during COVID-19	Discussion of online grocery shopping during COVID using other forms of payment than WIC	Discussion of the desire to be able to use WIC for online grocery shopping
Try new retailers	Shopping at different stores or food retailers than pre-COVID to try to avoid food shortages or other COVID-related barriers to accessing food	Discussion of trying new grocery stores or farmers markets to find food during the pandemic	Discussion of preferred stores for using WIC or general references to stores that have better supply of fruits or vegetables for CVB
Enrollment process changes	Perceptions, positive or negative, about changes to the process of WIC enrollment that occurred during COVID-19	Statements about feelings about changes to enrollment or WIC appointments that happened during/as a result of COVID such as phone appointments	Statements about desired changes to the enrollment process or feelings about the enrollment process prior to COVID-related changes
**WIC Enrollment**			
Enrollment process	Participant experiences with enrolling, certification, and education when first enrolling in WIC	Perceptions, positive or negative, about the WIC enrollment process and desired changes to WIC enrollment processes	Desired changes to WIC eligibility
Facilitators to enrollment	Positive aspects that helped participants enroll in WIC, can include processes that existed pre- or post-COVID	Family referrals, visits while in the hospital postpartum, remote appointments	Changes that would make it easier to enroll
Barriers to enrollment	Challenges to enrolling in WIC or remaining enrolled in WIC pre- or post-COVID	Limited staffing, missing WIC contact information, no reminders, in person appointments	Changes that would make it easier to enroll
Desired changes to enrollment	Elements of enrollment process that participants would like to see added or changed	Screening questions about mothers' weight, ways to improve discussions about household dietary preferences	Barriers to enrollment
Benefits of WIC enrollment	Other positive perceptions of being enrolled in WIC beyond the food package	Relationships with WIC staff, education offered, providing pumps, financial support/being able to save money that would have been used on food for other household needs	Benefits associated with the foods received through WIC
Drawbacks of WIC enrollment	Other negative feelings about being enrolled in WIC beyond the food package	Feeling judged by WIC staff, negative interactions with WIC staff, pressure to breastfeed	Drawbacks or negative perceptions about the food received through

			WIC, challenges that occur during the enrollment period
