# Supplementary material for: “I Think That’s the Most Beneficial Change That WIC Has Made in a Really Long Time”: Perceptions and Awareness of an Increase in the WIC Cash Value Benefit
Source: Int J Environ Res Public Health. 2022 Jul 16;19(14):8671. doi: 10.3390/ijerph19148671 (PMC9324401; doi:10.3390/ijerph19148671)
Supplement: Supplementary file 1 [file ijerph-19-08671-s001.zip › Supplemental Table S2.pdf]

Supplemental Table S2. Themes, subthemes, and demonstrative quotes.

Theme		Demonstrative Quote(s)
	Subtheme	
**Pre-COVID Cash Value Benefit**		
	Perceptions of amount	"It's not enough. It's not it's just like a bag of apples and a package of strawberries. I mean, what's that going to do? You know what I mean? Like it's nothing."
	Strategies to stretch Cash Value Benefit	"To a certain extent, it was just like, we just had to be more careful about the portion sizes and like pay attention, really like pay attention to the sizes like if he gets grapes and everything instead of like getting two pounds of grapes, we'll have to make sure like to get at least like one pound so he could get a vegetable to go along with the fruit."
**Awareness of Cash Value Benefit Changes**		
	Notified about changes	"I think my WIC, my WIC lady, the one that normally does the appointment, she kind of just called me like well you know you gettin' extra benefits. She just kind of made me aware of it."
	Not notified about changes	"I remember when we got the first increase, I wasn't told about it. So I called WIC to make sure that it was accurate because I didn't want to spend it and then have to be responsible for repaying it…"
	Challenges associated with October decrease	"Yeah, like going into the grocery store or whatever you get the receipt or whatever and you're like "Dang, zeroed out. How is it zeroed out?" Then you look on your benefit amount and it's like "Oh, we ain't get nowhere near as much," and it's like well…thank you EBT."
	Uncertainty about Cash Value Benefit amounts	"But yeah, like everybody else was saying, if it was something that we could rely on and was consistent, that would be better. But I mean, right now, it's kind of you just got to play it by ear, because you don't know what the changes are going to be."
**Perceptions of Cash Value Benefit Changes**		
	Achieve family's preferences	"…and I'm Hispanic. So we do use a lot of vegetables, you know, in like sauces or in guacamole or you know, just fresh. It's just so amazing. I have all that fresh fruit and vegetables on hand and make and make more with it. You know? And I'm not worried about it being gone in a week."
	Value of the Cash Value Benefit	"I will repeat that because I brought that up when I first spoke to someone the fruits and vegetables I think is like the most important thing. And I think that is more important than eggs, more important than milk, more important than cereal. They all have their benefits. But I think the vegetables, especially if you're going to start the kids off when they're young, you have to give them the vegetables when they're little or they're not going to want them."
	Dissatisfaction with amounts after November 2021	"The amounts we have, you know, since they're a little less of what we had in the summer, it does kind of limit what we can purchase."
**Cash Value Benefit Use Perceptions**		

	Barriers	"A lot of times they'll be out of a lot of things or the selection isn't really good. It's moldy or, or aged or old. So I kind of have to skip getting certain things because they're, they're not really worth buying."
	Facilitators	"Yeah there's so much you can get with it I don't know how you could not use it"
**Perceived Changes in Food Behaviors**		
	Variety	"With that increase in fruits and vegetables for my kids, I got to introduce them to vegetables that they would probably never eat at a young age. My child was asking me for asparagus all the time. And asparagus is expensive. Asking me for broccoli, like things that I would have never in 1000 years thought she would ask me for but she started asking me for this stuff."
	Child diet	"Yeah, like she said fruits and vegetables are expensive. So whenever I didn't have, you know, for fruits and vegetables, like whenever I would use my cash value benefits for my child's snacks, I would have to buy like cookies or something. So now they eat actually healthier."
	Caregiver or family diet	"I do remember munching on fruits more than like grabbing a $2 pack of cookies you know those pregnancy cravings how those kick in and my husband would buy more fruits rather than you know, buying me a big old bag of chips or cookies."
	Engaging children while shopping	"And he's getting to engage more in shopping and everything like that. So he's like "well, I want this" and "I want this fruit, or I want this vegetable", and everything like that. So it's giving him a chance to engage and eat healthier."
**WIC Utilization Barriers**		
	Checkout experience	"And it gets frustrating because sometimes I have $0, I have no money, and I'm relying solely on my WIC card. So you know, I'm utilizing the app and I'm putting things into my cart that I think is WIC eligible, but then I go and I get it rung up and they say you have a balance of $17.82 that I have to pay out of pocket and I'm like well "Whoa, this is all WIC stuff. How am I owing you know, cash?" So then we have to go through that frustrating task of trying to see what exactly did I get that wasn't eligible? Was this juice too big? Was this cheese not the right brand? And that can get frustrating when you have, you know, a long line of people behind you when we got to start doing all of that."
	Time or mental burden	"I know I often go same grocery store. Because I know like, like, if it doesn't change, I like know exactly where it is in the store. It's when I have to go to a different store that I get all confused because…because it is either marked differently or it's in a different section and so, yeah."
	Desire for new technology	"And so like I finished my shift at six in the morning, and when I go to Food Lion there no registers open. It's just self checkout. And so I have to like find somebody to open a register just to get my WIC stuff for the kids for breakfast or something. So yeah, being able to use self checkout would be really nice."
	Lack of labeling	"I don't ever go to Walmart either for WIC because usually they're not labeled or the label is a lot harder to find than it is that other grocery stores"
	Stigma	"If I tried to get the same products that I saw somebody else getting in the app, like I can't get it, it's not approved. And you know, I'm left embarrassed. I mean, you know, I gotta pay for it."
	Store environment	"Normally I just avoid Walmart altogether and would rather though I could get more for my dollar at Walmart, I just avoid it because the lines and it's just aggravating to use WIC there."

	BNFT app	"And I can definitely relate because when I go to the grocery store, I honestly the grocery store where I go to there's not a lot of phone service there. So I have to screenshot what benefits are available before I actually go to the store. Because I know once I get there, I'm not going to have any service to be able to check the app."
**WIC Utilization Facilitators**		
	Electronic Benefit Transfer (EBT) transition	"Using WIC is pretty easy now that they have switched over to the card instead of paper vouchers. And we can shop freely and just swipe the WIC card and whatever is eligible will be taken off."
	Labeling	"Well personally, I do side with everyone else that says Food Lion because Food Lion has a better variety of like WIC foods and it is also has labels that say it is WIC approved."
	Store environment	"I also like Food Lion and we don't have a Harris Teeter but we have a Lowe's Foods and they are really good. But also what I've seen at Food Lion and Lowe's foods is that the employees are also really kind and helpful."
	BNFT app	"Yes, we love that app. Because we did not have it when our when our boys were in it. And so I was like walking around with this huge book like trying to flip through it. The app is much nicer. Much easier."
**Food Access Barriers During COVID**		
	Inflation	"And also, with all the shortages that are happening with fruits and vegetables, and sometimes the price increase, I'm like, well, WIC should think, okay, if the prices of fruits and vegetables, and all the other stuff are going up, shouldn't WIC also up their [inaudible] to match that? Because it doesn't make sense if WIC is giving you a certain amount of money to buy some stuff, you can only get once like, it doesn't make sense. WIC should match whatever the benefits are to how we're living today…"
	Shortages	"Like last month my problem was finding formula for him. Because there was I guess a formula shortage. And I would have to be literally traveling for two hours all around North Carolina checking calling Wal-Mart to see if they have formulas available for him."
	In-person shopping and risk	"And online you can't really use WIC you know they, they added the EBT but you can't, they didn't add WIC up there. So it was more risky as for me to go inside and get the groceries. But like I couldn't really you know stand in the aisle and ponder "hmm, which can of beans do I want today." I really had to like grab and go. You know, it was, it was scary. It still is scary out there. But it's gotten better. Yeah, it was difficult during the pandemic."
**Food Access Facilitators During COVID**		
	WIC flexibilities	"But recently, during the shortages, they started allowing us to get 2% which has helped because again, a lot of this stuff is out of stock and it's continuously like I'll go to multiple stores and I can't find the organic skim milk, it's always out. So when they loosened the restrictions up a little bit and let us choose like a 2% option that helped tremendously."
	Online shopping with other payment types	"AmazonFresh. Being able to use that, well not for WIC, but for EBT. That was great."
**Desired Changes to WIC Food Package**		

	Cash Value Benefit changes	"So I wish that they would continue to give us the fruits and vegetable money once they turned six months. So that I could do that without having to spend you know my own money or I mean I don't mind spending my own money but it helps having you know the WIC to cover it but because she loves fresh fruits and vegetables as well."
	Amount	"So all these gallons of milk that you give me like I don't need six gallons of milk for two kids. Like, I definitely need more eggs for two kids because they eat through that. And I definitely need more fruits and vegetables because they eat through that and more cereals..."
	Eligibility	"And then when you do breastfeed, they cut you off at a year. And there's moms who go past a year, so we don't get they cut, they don't allow the moms to have the packages after the year. And there's moms who breastfeed past a year."
	Flexibility	"I think that it would be a positive turn for WIC to start thinking about families who have alternative diets. You know families who, you know, don't, don't eat dairy or because that, that I hate that that stuff goes to waste."
	Rolling over benefits	"…with WIC I wish that a lot of the stuff would roll over. Like, like I said previously…certain things like with the eggs, my child doesn't eat them all the time. And then she turns around, and she's scarfs 'em down and sometimes I don't always get food stamps."
